# Assessing the Impact of Sociodemographic Factors on Artificial Intelligence Models in Predicting Dementia: Retrospective Cohort Study

**DOI:** 10.2196/80405

**Published:** 2026-02-17

**Authors:** Xingyi Liu, Muskan Garg, Maria Vassilaki, Jennifer St Sauver, Ronald C Petersen, Momin M Malik, Chung Il Wi, Young J Juhn, Sunghwan Sohn

**Affiliations:** 1 Department of Artificial Intelligence and Informatics, Mayo Clinic, Harwick Building, 7th Fl., 205 3rd Ave SW, Rochester, MN, 55905, United States, 1 507-266-0376; 2 Department of Quantitative Health Sciences, Mayo Clinic, Rochester, MN, United States; 3 Department of Neurology, Mayo Clinic, Rochester, MN, United States; 4 Department of Pediatric and Adolescent Medicine, Mayo Clinic, Rochester, MN, United States

**Keywords:** artificial intelligence, AI bias, dementia, Area Deprivation Index, socioeconomic status, machine learning

## Abstract

**Background:**

Artificial intelligence (AI) is increasingly applied to health care, yet concerns about fairness persist, particularly in relation to sociodemographic disparities. Previous studies suggest that socioeconomic status (SES) and sex may influence AI model performance, potentially affecting groups that are historically underserved or understudied.

**Objective:**

This study aimed to (1) assess algorithmic bias in AI-driven dementia prediction models based on SES and sex (biological sex), (2) compare the utility of an individual-level SES measure (the Housing-Based Socioeconomic Status [HOUSES] Index) versus an area-level measure (the Area Deprivation Index) for bias detection, and (3) evaluate the effectiveness of an oversampling technique (the Synthetic Minority Oversampling Technique for Nominal and Continuous features) for bias mitigation.

**Methods:**

This study used data from two population-based cohorts: the Mayo Clinic Study on Aging (n=3041) and the Rochester Epidemiology Project (n=19,572). Four AI models (random forest, logistic regression, support vector machine, and Naïve Bayes) were trained using a 5-year observation window of structured electronic health record data to predict dementia onset within the subsequent 1-year window. Fairness and model performance were assessed using the balanced error rate (BER) across intersecting SES-sex subgroups. The Synthetic Minority Oversampling Technique for Nominal and Continuous features algorithm was applied to the training data to balance the representation of SES groups.

**Results:**

Across both cohorts, individuals with lower SES generally exhibited higher BERs (worse performance) than high SES groups, confirming the presence of bias. In the MCSA cohort, males with high SES, as indicated by the HOUSES Index, consistently exhibited the lowest BERs across all evaluated models. Balancing the training data based on a specific SES measure showed a trend toward reducing the BER disparity when evaluated using that same measure. However, this targeted improvement demonstrated nonuniversal benefits; in some cases, it exacerbated disparities when evaluated using other, unbalanced SES measures. This pattern suggests that fairness interventions are not universally beneficial across different definitions of the protected attribute. While the balancing approach improved fairness in model performance for lower SES groups, it often came at the cost of a slight reduction in overall model performance. However, an exception was observed in the MCSA cohort when balancing based on the HOUSES Index using logistic regression, support vector machine, and Naïve Bayes, where the performances of both the high and low SES groups improved.

**Conclusions:**

This research highlights the importance of incorporating sociodemographic context into AI modeling in health care. The choice of SES measure may lead to different assessments of algorithmic bias. The HOUSES Index, as a validated individual-level SES measure, may be more effective for bias mitigation than area-level measures. Future AI development should integrate bias mitigation strategies to ensure models do not reinforce existing disparities in health outcomes.

## Introduction

In 2024, Alzheimer dementia affected approximately 6.9 million people in the United States, and this number is projected to escalate to 13.8 million by 2060. This growth places an immense burden on patients and their families, society, and the health care system, manifesting in an estimated 18.4 billion hours of unpaid care annually, valued at US $346.6 billion [1]. Despite this considerable prevalence, cognitive impairment, including mild cognitive impairment (MCI) and dementia, often goes undetected in clinical settings. Diagnoses frequently come late in the cognitive decline process, reducing opportunities to enhance patients’ quality of life and exacerbating existing health disparities. Ongoing research in health care seeks to leverage artificial intelligence (AI) to enhance dementia prediction.

The rapid advancements in computing power, data storage, and predictive analytics have significantly accelerated the integration of AI tools into the US health care system [2]. By 2014, 97% of nonfederal acute care hospitals had adopted a certified electronic health record (EHR) [3]. As of January 2025, the US Food and Drug Administration has approved more than 1000 clinical AI applications [4]. This foundational shift has enabled the widespread adoption of AI. AI-powered solutions have already demonstrated transformative potential in health care.

Beyond diagnostics, AI research continues to explore ways to address health disparities, such as reducing unexplained pain disparities through image-based algorithms in underserved populations [5]. However, alongside these advancements lies the critical challenge of mitigating biases inherent in AI systems. Studies have revealed that AI algorithms trained on biased data can perpetuate health disparities, particularly among underresourced and understudied populations [6-9]. For example, a race-based algorithm for estimating kidney function produced higher estimates for Black patients than for White patients, resulting in delayed referrals for organ transplants among Black individuals [10]. Such findings underscore the ethical imperative to scrutinize differential model performance by socioeconomic status (SES) and other social determinants of health (SDOH), which have profound implications for underserved populations and care delivery. SES, as a core component of SDOH, plays a pivotal role in shaping health outcomes through biological, behavioral, and environmental pathways [11-20].

Recent studies have highlighted the pervasive issue of bias in health care AI systems, particularly SES. For instance, a 2019 study [21] defines algorithmic bias in health care as the application of an algorithm that compounds the challenges affecting underserved groups across SES, race, background, religion, sex, or disability, thereby amplifying disparities in health systems. The authors discuss how AI can inadvertently perpetuate and exacerbate challenges faced by underserved populations if not carefully designed and implemented. Another study outlines various elements of potential bias in the development and implementation of AI algorithms in health care. It emphasizes that such biases can propagate stereotypes and discrimination, further marginalizing underserved populations and contributing to socioeconomic health care disparities [22]. Similarly, research indicates that lower SES is associated with poorer predictive model performance, potentially due to incomplete or inaccurate EHR data [23].

In addition to SES disparities, sex differences have emerged as a critical factor influencing AI model performance in dementia prediction. Research indicates that females often exhibit more severe cognitive impairment and experience a faster rate of cognitive decline compared to males at the onset of Alzheimer disease [24]. Sociodemographic factors, such as lower education levels and SES, disproportionately affect older female individuals, increasing their risk for dementia [25-27]. Predictive models for cognitive decline, such as Cox regression, demonstrate varying performances across sexes, indicating potential biases in predictive accuracy [28]. Additionally, transfer learning approaches have revealed that cognitive deficits in female individuals, once detected at the MCI stage, tend to deteriorate more consistently over time, whereas male participants exhibit a wider variety of declines across multiple cognitive functions [29].

Despite these findings, there remains a significant gap in strategies to mitigate biases in AI models. While some studies have proposed methods to detect and quantify such biases, comprehensive approaches to address and correct them are limited [30]. Our study seeks to fill this gap by not only examining the relationship between SES, sex, and AI model performance in dementia prediction but also implementing oversampling techniques to ensure adequate representation of socioeconomically disadvantaged groups. By doing so, we aim to reduce disparities in model performance across different SES groups, thereby enhancing fairness in AI-driven health care solutions.

## Methods

### Study Population

In our study, we used 2 cohorts. First, we studied individuals participating in the Mayo Clinic Study on Aging (MCSA) [31]. The MCSA, which commenced in 2004, is a population-based research study focused on investigating the epidemiology of MCI, dementia, and related biomarkers (N=5890; dementia, n=682, 11.6%; and non-dementia, n=5208, 88.4%). Participants for the study were chosen randomly from among persons living in Olmsted County, Minnesota, at the time of recruitment. These participants completed detailed cognitive assessments, including the Clinical Dementia Rating scale, a full-scale neurological evaluation, and various neuropsychological tests. Using established criteria, a consensus committee made diagnoses, classifying participants as cognitively unimpaired, having MCI, or having dementia. MCI diagnostic criteria have been disclosed in previous publications [32], and dementia diagnoses adhered to the criteria laid out in the *Diagnostic and Statistical Manual of Mental Disorders, Fourth Edition* [33].

Second, we studied a larger cohort of persons living in Olmsted County, MN, between 2004 and 2020, derived from the Rochester Epidemiology Project (REP) medical records linkage system (N=290,528; dementia, n=8,205, 2.8%; and non-dementia, n=282,323, 97.2%) [34]. The onset of dementia was identified by the date of the first diagnosis code (*International Classification of Diseases [ICD], Ninth or Tenth Revision code*) received between 2004 and 2020.

### Socioeconomic Measures

We assessed potential biases in model performance using two residence-based SES measures: the Area Deprivation Index (ADI) [35] and the Housing-Based Socioeconomic Status (HOUSES) Index [36]. The ADI is a multidimensional evaluation of a person’s socioeconomic conditions at the census block group level. We used both national-level and state-level ADI rankings. The national-level ADI rankings were used to divide participants into two groups: those in the higher ADI quartile group (76-100) were considered to reside in an area with high socioeconomic deprivation (hereafter, low SES), and those in the ADI group (0‐75) were considered the referent group (hereafter, high SES). Similarly, the state-level ADI ranking was categorized into two groups: ADI (8-10) for low SES and ADI (0‐7) for high SES. The HOUSES Index is a housing-based SES indicator, derived from an analysis of 4 specific variables related to a housing unit. The bottom quartile was considered low SES, and the remaining quartiles were considered high SES. The HOUSES Index was only used in the MCSA cohort because it is not available for the REP cohort. By incorporating both the HOUSES Index and ADI measures, our study aimed to evaluate and compare their effectiveness in identifying SES-related biases in the performance of AI models. In addition to quantifying bias in model performance based on SES, our study also considered sex.

### Prediction Timeline Window

Our goal was to use records from the past 5 years (observation window) to forecast whether a patient would be diagnosed with dementia in the following year (prediction window).

**Figure 1.**
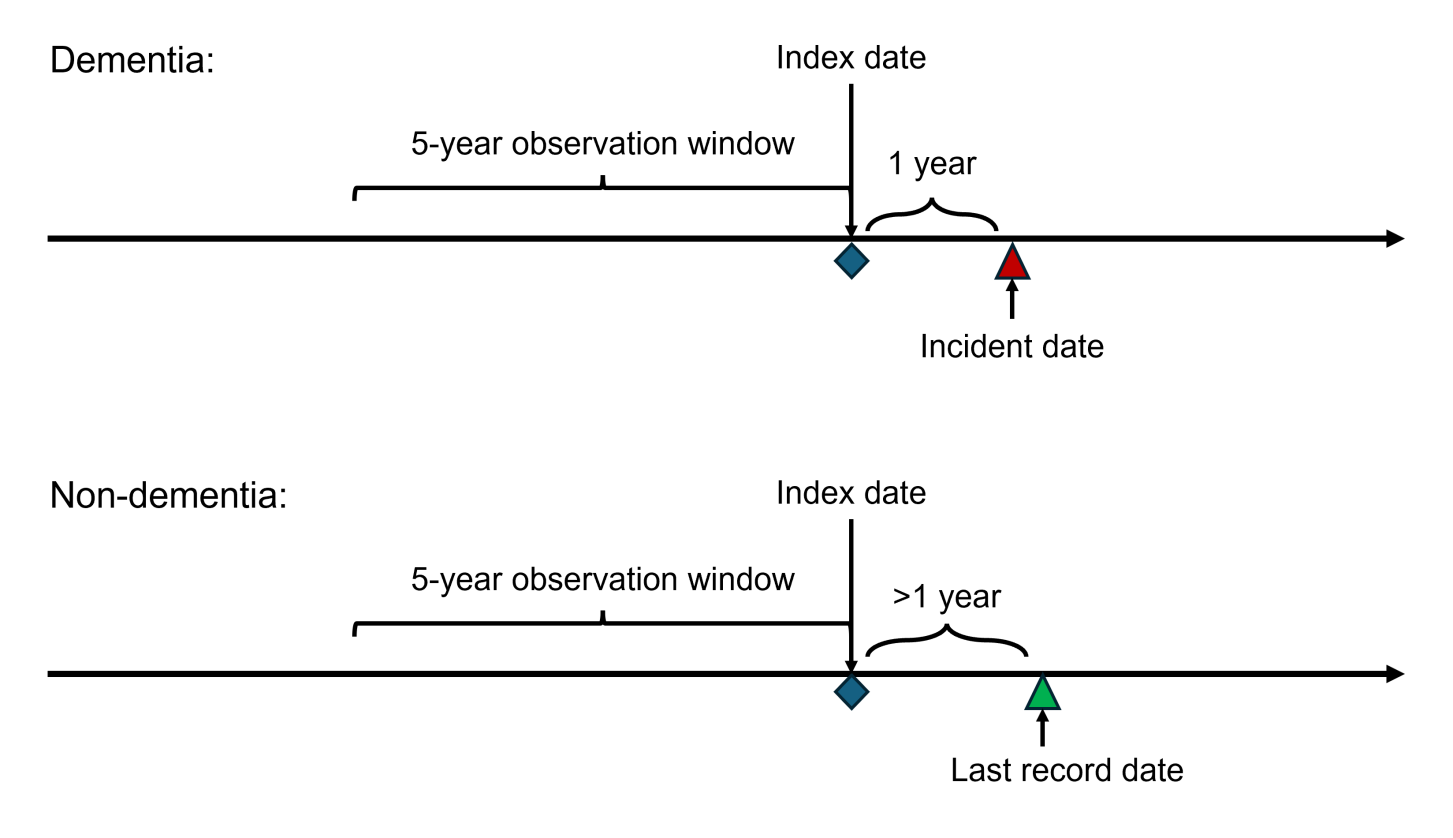
Timeline overview for dementia and non-dementia patients. For patients with dementia (cases), the index date is set 1 year before their diagnosis. For patients without dementia (controls), the index date is assigned to match the age distribution of the cases. The model uses a 5-year observation window preceding the index date to predict the onset of dementia within the subsequent 1-year prediction window.

For both cohorts, we applied consistent inclusion and exclusion criteria. For patients with dementia (case), the incident date was the date of dementia onset. We excluded patients whose age at the incident date was less than 50 years. The index date (point for prediction) is set to 1 year before the dementia incident date. We performed frequency matching for age between participants with dementia and participants without dementia. For patients without dementia (control), we assigned index dates to match age with cases—that is, patients without dementia at the index date have a similar age distribution to patients with dementia at their index dates. As shown in Figure 1, the 5-year period preceding the index date serves as the observation window. The 1-year period following the index date is the prediction window for both groups. We exclude patients with no record within the observation window. We also exclude patients without dementia who do not have at least 1 year of follow-up after their index date (whose last record date is not at least 1 year after the index date).

### Assessing AI Bias Across Different SES and Sex Categories

Four machine learning algorithms were selected to capture a variety of model classes commonly used in clinical prediction tasks, allowing for an assessment of whether observed bias patterns were robust across different algorithmic assumptions and complexities. The selected models were (1) logistic regression (LR), a well-established, interpretable linear model that serves as a standard baseline in clinical research; (2) support vector machine (SVM), a powerful kernel-based method capable of learning complex, nonlinear decision boundaries; (3) random forest (RF), a nonlinear ensemble of decision trees that is robust to overfitting and can implicitly capture complex interactions between features; and (4) Naïve Bayes (NB), a simple yet efficient probabilistic classifier based on Bayes’ theorem with strong independence assumptions. This selection enables a more comprehensive evaluation of how algorithmic bias manifests across linear, nonlinear, ensemble, and probabilistic modeling paradigms. We trained and tested the 4 AI models to predict the incidence of dementia in the following year.

The experimental process begins with preprocessing the data. The 5-year observation period is divided into five 1-year windows. During each 1-year window, patients may have multiple visits. For each visit, we extracted ICD-9 and ICD-10 codes, categorized the codes using the Clinical Classifications Software (CCS) [37], which groups diagnoses and procedures into clinically relevant categories and then generated a one-hot–encoded vector representing the CCS categories for the visit. We computed the element-wise sum of all visit vectors within each 1-year window. To robustly distinguish between years with no visit and years with visits but no relevant diagnoses, we used a missing indicator method. For 1-year windows where a patient had no visits, the corresponding annual vector was filled with zeros. Concurrently, we introduced 5 binary features, one for each year of the observation window, setting the feature to “1” to explicitly flag a year with no visits and “0” otherwise. Finally, we formed the final input vector by concatenating the 5 annual vectors, sex, age at the index date, and the 5 created binary visit-indicator features. Our primary objective was to assess the baseline performance and bias of models built only on the most common, structured data elements that are widely available for nearly all patients within a standard EHR system.

In this study, we developed and evaluated 4 AI models for each cohort. We randomly partitioned the dataset into two subsets: 70% for training and 30% for testing. Within the training set, we used a 5-fold cross-validation strategy to identify the optimal parameters for each model, thereby ensuring robust performance estimates and minimizing the risk of overfitting. After determining the best-performing configuration for each model, we retrained the model on the entire training set and subsequently evaluated its performance using the 30% test set. All model development and evaluation procedures were conducted independently within each of the 2 cohorts. We did not perform cross-cohort validation.

Within each cohort, and for each model, we then conducted subgroup analyses by comparing performance across different sex groups and among various SES-sex subgroups. This approach was used to identify potential differences in predictive bias.

To assess the fairness of these models, we used the balanced error rate (BER) [23], defined as the unweighted mean of the false-positive rate (FPR) and the false-negative rate (FNR), as follows:


$$BER=\frac{FPR+FNR}{2}$$

The FPR, also referred to as 1−specificity, represents the proportion of negative instances that are incorrectly classified as positive. Formally,


$$FPR=\frac{False\;positive(FP)}{True\;negative(TN)+False\;positive(FP)}$$

The FNR, also referred to as 1−sensitivity, represents the proportion of positive instances that are incorrectly classified as negative. Formally,


$$FNR=\frac{False\;negative(FN)}{True\;positive(TP)+False\;negative(FN)}$$

By incorporating both the FPR and FNR, BER captures imbalances in how models treat positively and negatively labeled individuals, allowing us to identify potential disparities or biases in the predictive performance.

To assess the variability and reliability of the BER estimates, we calculated bootstrap CIs for each SES-sex group:

1. Bootstrapping procedure: We generated 1000 bootstrap samples by randomly resampling with replacement from the original test datasets.
2. BER calculation: For each bootstrap sample, we calculated the BER.
3. CI estimation: The distribution of the bootstrap BERs was used to compute the 95% CI, providing an estimate of the uncertainty around the BER.

To assess statistical differences in model performance (BER) between SES-sex groups, we conducted a series of pairwise 2-tailed *t* tests with a significance threshold of 0.05. The Levene test was used to assess variance homogeneity, with the Welch *t* test applied for unequal variances and the standard *t* test for equal variances. Each test was performed independently to evaluate a specific contrast (eg, low vs high SES within a sex group). To correct for the increased risk of type 1 errors (false positives) due to multiple comparisons, we applied the Benjamini-Hochberg procedure. For each of the 4 AI models, we collected the *P* values from all pairwise *t* tests performed for that model and applied the Benjamini-Hochberg correction to this complete set of *P* values. This method controls the false discovery rate at our chosen significance level of 0.05. All reported statistical significances for pairwise comparisons are based on the results of this false discovery rate correction.

In addition to these pairwise comparisons, a separate baseline analysis was conducted to contextualize the performance of all subgroups relative to a single reference point. For this analysis, the “male in high SES” subgroup was designated as the baseline category. We then calculated the difference in BER for all other sociodemographic subgroups by comparing their BERs to the BER of this baseline group. This method provides a consistent benchmark for evaluating the performance disparities relative to the reference group.

### Reducing SES-Related Bias

To address disparities in AI model performance related to SES, we implemented the Synthetic Minority Oversampling Technique for Nominal and Continuous features (SMOTE-NC) [38]. SMOTE-NC is a variant of an oversampling technique designed to generate synthetic examples in datasets that include both continuous and categorical variables. This method is particularly useful in addressing imbalances in datasets, which can lead to biased model performance.

In the context of our study, we applied SMOTE-NC to balance the representation of understudied SES groups within our training dataset. This process preserved the ratio of dementia cases and controls in the cohort (Tables 1 and 2). We set the number of neighbors parameter to 5 and used a fixed random seed of 42. This process ensured that the distribution of SES groups in the training dataset was balanced, without introducing synthetic examples into the test set. Following the oversampling, the AI models were retrained on the balanced dataset using the same cross-validation strategy. To assess the effectiveness of SES balancing, we compared the performance (BER) of models trained on the oversampled datasets with those trained on the original, imbalanced datasets.

**Table 1.** Participant characteristics in Mayo Clinic Study on Aging (MCSA; N=3041), stratified by dementia cases (n=679, 22.3%) and non-dementia controls (n=2362, 77.7%).

MCSA	Case	Control
Age (y; at index date), n (%)		
55‐64	1 (0)	3 (0)
65‐74	32 (5)	112 (5)
75‐84	259 (38)	906 (38)
85‐94	364 (54)	1261 (53)
≥95	23 (3)	80 (4)
Sex, n (%)		
Male	353 (52)	1192 (51)
Female	326 (48)	1170 (49)
Race, n (%)		
Native American or Alaska Native	0 (0)	0 (0)
Asian	8 (1)	11 (1)
Hawaiian or Pacific Islander	0 (0)	1 (0)
Black or African American	1 (0)	5 (0)
White	666 (98)	2337 (99)
More than one	3 (1)	5 (0)
Missing	1 (0)	3 (0)
Background, n (%)		
Hispanic	2 (0)	6 (0)
Non-Hispanic	674 (99)	2350 (100)
Missing	3 (1)	6 (0)
HOUSES^a^ Index, n (%)		
Q1 (low SES^b^)	146 (22)	506 (21)
Q2-Q4 (high SES)	533 (78)	1856 (79)
National-level ADI^c^, n (%)		
76‐100 (low SES)	35 (5)	94 (4)
0‐75 (high SES)	644 (95)	2268 (96)
State-level ADI, n (%)		
8‐10 (low SES)	202 (30)	572 (24)
0‐7 (high SES)	477 (70)	1790 (76)
Number of visits, mean (SD)	93.3 (65.0)	79.5 (62.7)

a HOUSES: Housing-Based Socioeconomic Status.

b SES: socioeconomic status.

c ADI: Area Deprivation Index.

**Table 2.** Participant characteristics in Rochester Epidemiology Project (REP; N=19,572), stratified by dementia cases (n=7335, 37.5%) and nondementia controls (n=12,237, 62.5%).

REP	Case	Control
Age (y; at index date), n (%)		
50‐54	97 (1)	194 (1)
55‐64	405 (6)	810 (7)
65‐74	1199 (16)	2398 (20)
75‐84	2830 (39)	5660 (46)
85‐94	2485 (34)	3016 (25)
≥95	319 (4)	159 (1)
Sex, n (%)		
Male	3040 (41)	5298 (43)
Female	4295 (59)	6939 (57)
Race, n (%)		
Black	80 (1)	123 (1)
Asian	118 (2)	229 (2)
Hawaiian or Pacific Islander	3 (0)	4 (0)
American Indian	3 (0)	11 (0)
Other or mixed	58 (1)	121 (1)
White	7049 (96)	11,681 (96)
Refusal	11 (0)	24 (0)
Unknown	13 (0)	44 (0)
Background, n (%)		
Hispanic	105 (1)	238 (2)
Non-Hispanic	6504 (89)	10,536 (86)
Missing	726 (10)	1463 (12)
National-level ADI^a^, n (%)		
76‐100 (low SES^b^)	444 (6)	651 (5)
0‐75 (high SES)	6891 (94)	11,586 (95)
State-level ADI, n (%)		
8‐10 (low SES)	2098 (29)	3176 (26)
0‐7 (high SES)	5237 (71)	9061 (74)
Number of visits, mean (SD)	76.2 (70.4)	55.8 (52.2)

a ADI: Area Deprivation Index.

b SES: socioeconomic status.

### Ethical Considerations

The study was approved by the institutional review boards (IRBs) of the Mayo Clinic (20‐004498) and the Olmsted Medical Center (028-OMC-20). The need for informed consent was waived for the study. Approval for the MCSA (IRB number 14‐004401) was also obtained from the IRBs of the Mayo Clinic and Olmsted Medical Center in Rochester, Minnesota. Participants of the MCSA provided written informed consent before participation. The MCSA informed consent and the study’s IRB protocol allow secondary analysis without additional consent. All data were accessed and analyzed within secure and institutionally approved computing environments. No individual identifiers were presented in the manuscript. The results are reported only in aggregate form, ensuring that patients cannot be identified. No compensation was provided to participants, as this study involved the retrospective analysis of medical records.

## Results

### Cohort Characteristics

Table 1 presents the participant characteristics for the MCSA cohort. Approximately 21% (652/3041) of cases were categorized as low SES based on the HOUSES Index, 4% (129/3041) were categorized based on the national-level ADI, and 25% (774/3041) were categorized based on the state-level ADI. To understand the relationship between SES measures, we assessed the concordance between the HOUSES Index and both ADI measures in the MCSA cohort. There was limited overlap between the classifications. Among those identified as low SES by the HOUSES Index, only 12.0% (78/652) were concurrently classified as low SES by the national-level ADI, and 40.0% (261/652) were concurrently classified as low SES by the state-level ADI. This lack of concordance indicates that while these metrics all aim to measure disadvantage, they identify distinct subpopulations.

Table 2 summarizes the participant characteristics for the REP cohort. SES analyses indicate that approximately 6% (1095/19,572) of cases were classified as low SES using the national-level ADI, and 27% (5274/19,572) of cases were classified as low SES using the state-level ADI (the HOUSES Index is not available in the REP cohort). SES measure distributions were similar for controls in both populations.

The average number of visits during the 5-year observation window was 93.3 (SD 65.0) for patients with dementia and 79.5 (SD 62.7) for patients without dementia in the MCSA cohort, and 76.2 (SD 70.4) and 55.8 (SD 52.2), respectively, in the REP cohort. We also assessed data completeness by calculating the average number of years missed, defined as the count of years within the 5-year observation window where a patient had no recorded visits. When analyzing the combined population of patients with dementia and patients without dementia, most SES measures indicated that the low SES group exhibited a higher average number of years missed compared to the high SES group. In the MCSA cohort, the high SES group averaged 0.12 missed years compared to 0.17 missed years for the low SES group based on the HOUSES Index. Similarly, the national-level ADI showed 0.13 missed years for the high SES group and 0.16 missed years for the low SES group, while the state-level ADI showed a divergent pattern, with 0.14 missed years for the high SES group compared to 0.11 missed years for the low SES group. A consistent pattern emerged in the REP cohort, where the national-level ADI showed averages of 0.54 missed years for the high SES group and 0.71 for the low SES group, and the state-level ADI showed 0.54 for the high SES group compared to 0.59 for the low SES group.

**Figure 2.**
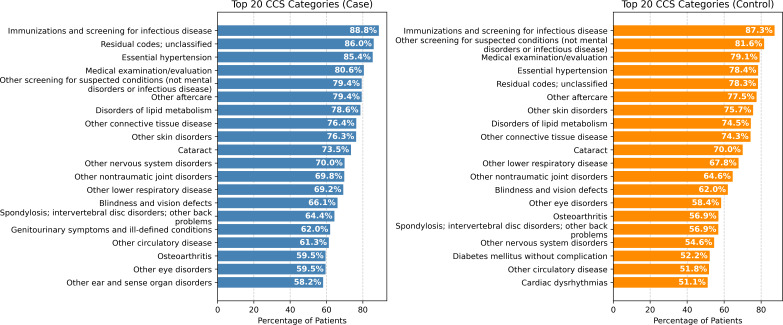
Top 20 Clinical Classifications Software (CCS) categories that appear among cases versus controls during the 5-year observation period, ordered by the percentage of patients who had those CCS categories in the Mayo Clinic Study on Aging cohort.

Figures2  3 show the top 20 CCS categories that appear among cases versus controls, ordered by the percentage of patients who had those CCS categories in the MCSA cohort and the REP cohort, respectively. In both cohorts, more patients with dementia visited for “essential hypertension” than patients without dementia during the 5-year observation period (MCSA: 85.4% vs 78.4%; and REP: 76.2% vs 67.0%). Similarly, more patients with dementia visited for “other nervous system disorders” than patients without dementia during the same period (MCSA: 70.0% vs 54.6%; and REP: 61.3% vs 38.4%).

**Figure 3.**
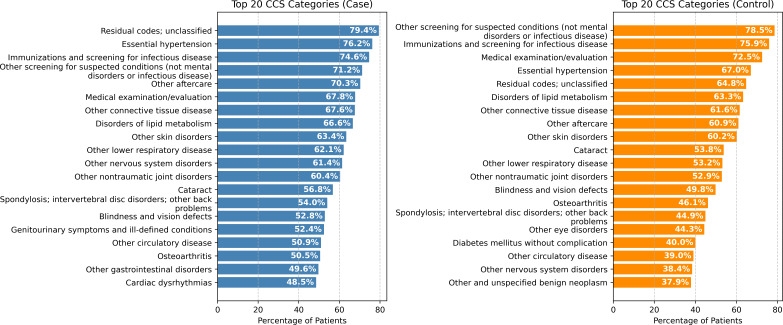
Top 20 Clinical Classifications Software (CCS) categories that appear among cases versus controls during the 5-year observation period, ordered by the percentage of patients who had those CCS categories in the Rochester Epidemiology Project cohort.

### Bias Measurement

Table 3 presents a comparison of BERs for AI-based dementia prediction models stratified by SES, as indicated by both the ADIs and the HOUSES Index in the MCSA cohort. The first 2 rows compare BERs among males, the next 2 rows compare BERs among females, and the final 2 rows compare BERs for both males and females combined. Between each pair of rows, superscript letters denote statistically significant differences in BER for the low SES group compared to the high SES group (based on Benjamini-Hochberg–corrected *P*<.05): “h” indicates a significantly lower BER, “i” indicates a significantly higher BER, and “j” indicates no statistically significant difference. Across all evaluated models, individuals (males and females combined) with low SES, as measured by both the ADIs and the HOUSES Index, consistently exhibited higher BERs. When focusing on males only, those with low SES similarly demonstrated higher or equal BERs across models. However, for females, the patterns differed: in nearly all evaluated models, females with low SES, as indicated by the national-level ADI, consistently showed higher BERs, while female participants with low SES, as indicated by the state-level ADI, showed comparable BERs. In contrast, those with low SES as measured by the HOUSES Index consistently exhibited lower or equal BERs.

Table 4 compares BERs within each AI-based dementia prediction model among SES-sex subgroups in the MCSA cohort. We selected “male in high SES” as the baseline category. For each model, the table displays how the BERs for “male in low SES,” “female in high SES,” and “female in low SES” compare to this baseline. “Lower” indicates a lower BER than the baseline, “higher” indicates a higher BER, and empty cells signify no statistically significant difference. From the table, it is evident that males with high SES, as indicated by the HOUSES Index, consistently exhibited the lowest BERs across all evaluated models. Furthermore, males with high SES measured by the 2 ADIs showed the lowest BERs in almost all models, with the exception of the RF when using the national-level ADI and the NB model when using either the state-level or national-level ADI.

Table 5 presents a comparison of BERs for AI-based dementia prediction models stratified by SES—as indicated by both ADIs—in the REP cohort (the HOUSES Index was not available in the REP cohort). In every evaluated model except NB, individuals (combining both males and females) with low SES, as determined by both ADIs, consistently exhibited higher BERs. When focusing on females only, those with low SES similarly demonstrated higher BERs across almost all models, with the exception of the NB model when using the state-level ADI. Furthermore, males with high SES showed lower BERs in almost all models, with the exception of the NB model when using either the state-level or national-level ADI.

**Table 3.** Comparison of balanced error rates (BERs) for artificial intelligence–based dementia prediction models in the Mayo Clinic Study on Aging (MCSA) cohort.^a^

MCSA (sex and SES group)	National-level ADI^m^, BER (95% CI)				State-level ADI, BER (95% CI)				HOUSES^c^ Index, BER (95% CI)			
	RF^d^	LR^e^	SVM^f^	NB^g^	RF	LR	SVM	NB	RF	LR	SVM	NB
Male												
High	0.2827^h^ (0.23-0.33)	0.3598^h^ (0.30-0.41)	0.4419^h^ (0.40-0.48)	0.4207^h^ (0.37-0.47)	0.2546^h^ (0.20-0.31)	0.3368^h^ (0.28-0.40)	0.4334^h^ (0.39-0.48)	0.4151^h^ (0.36-0.47)	0.2688^h^ (0.22-0.32)	0.3404^h^ (0.28-0.40)	0.4273^h^ (0.38-0.47)	0.3907^h^ (0.33-0.44)
Low	0.5408^i^ (0.17-0.88)	0.5378^i^ (0.21-0.88)	0.4531^i^ (0.12-0.67)	0.5247^i^ (0.20-0.80)	0.3957^i^ (0.29-0.50)	0.4482^i^ (0.34-0.56)	0.4651^i^ (0.38-0.54)	0.4447^i^ (0.34-0.54)	0.3767^i^ (0.26-0.49)	0.4591^i^ (0.33-0.58)	0.4996^i^ (0.44-0.54)	0.5708^i^ (0.50-0.63)
Female												
High	0.3157^i^ (0.26-0.37)	0.3661^h^ (0.31-0.42)	0.4790^h^ (0.44-0.51)	0.3822^h^ (0.33-0.43)	0.3053^h^ (0.24-0.37)	0.3709^j^ (0.31-0.43)	0.4745^i^ (0.44-0.51)	0.3867^h^ (0.33-0.45)	0.3269^i^ (0.26-0.39)	0.3775^i^ (0.31-0.45)	0.4908^i^ (0.45-0.53)	0.3964^i^ (0.33-0.46)
Low	0.2419^h^ (0.10-0.42)	0.4646^i^ (0.27-0.67)	0.5147^i^ (0.36-0.64)	0.5436^i^ (0.33-0.72)	0.3221^i^ (0.23-0.42)	0.3715^j^ (0.27-0.47)	0.4424^h^ (0.36-0.52)	0.4104^i^ (0.31-0.51)	0.2750^h^ (0.18-0.38)	0.3573^h^ (0.25-0.46)	0.4555^h^ (0.38-0.52)	0.3921^h^ (0.29-0.49)
Male and female												
High	0.3006^h^ (0.26-0.34)	0.3625^h^ (0.32-0.40)	0.4586^h^ (0.43-0.48)	0.4036^h^ (0.37-0.44)	0.2784^h^ (0.24-0.32)	0.3525^h^ (0.31-0.39)	0.4537^h^ (0.39-0.51)	0.4012^h^ (0.36-0.45)	0.2943^h^ (0.25-0.34)	0.3579^h^ (0.32-0.40)	0.4558^h^ (0.42-0.49)	0.3925^h^ (0.35-0.43)
Low	0.3258^i^ (0.18-0.49)	0.4844^i^ (0.31-0.66)	0.4902^i^ (0.35-0.62)	0.5437^i^ (0.37-0.69)	0.3627^i^ (0.29-0.44)	0.4074^i^ (0.34-0.48)	0.4620^i^ (0.43-0.49)	0.4292^i^ (0.36-0.50)	0.3181^i^ (0.24-0.39)	0.3971^i^ (0.32-0.48)	0.4721^i^ (0.42-0.52)	0.4617^i^ (0.39-0.53)

a Superscript alphabets indicate a statistically significant difference in BER for the low socioeconomic status (SES) group compared to the high SES group within that stratum, based on Benjamini-Hochberg–corrected *P*<.05.

b ADI: Area Deprivation Index.

c HOUSES: Housing-Based Socioeconomic Status.

d RF: random forest.

e LR: logistic regression.

f SVM: support vector machine.

g NB: Naïve Bayes.

h BER significantly lower.

i BER significantly higher.

j No statistically significant difference.

**Table 4.** Balanced error rate (BER) difference for male in high socioeconomic status (SES) as a baseline for Table 3.

MCSA^a^ (sex and SES group)	National-level ADI^b^, BER				State-level ADI^b^, BER				HOUSES^c^ Index, BER			
	RF^d^	LR^e^	SVM^f^	NB^g^	RF	LR	SVM	NB	RF	LR	SVM	NB
Male												
High	Base	Base	Base	Base	Base	Base	Base	Base	Base	Base	Base	Base
Low	Higher^h^	Higher^h^	Higher^h^	Higher^h^	Higher^h^	Higher^h^	Higher^h^	Higher^h^	Higher^h^	Higher^h^	Higher^h^	Higher^h^
Female												
High	Higher^h^	Higher^h^	Higher^h^	Lower^i^	Higher^h^	Higher^h^	Higher^h^	Lower^i^	Higher^h^	Higher^h^	Higher^h^	Higher^h^
Low	Lower^i^	Higher^h^	Higher^h^	Higher^h^	Higher^h^	Higher^h^	Higher^h^	Lower^i^	Higher^h^	Higher^h^	Higher^h^	—^j^

a MCSA: Mayo Clinic Study on Aging.

b ADI: Area Deprivation Index.

c HOUSES: Housing-Based Socioeconomic Status.

d RF: random forest.

e LR: logistic regression.

f SVM: support vector machine.

g NB: Naïve Bayes.

h Higher: BER significantly higher than baseline.

i Lower: BER significantly lower than baseline.

j No statistically significant difference from baseline.

**Table 5.** Comparison of balanced error rates (BERs) for artificial intelligence–based dementia prediction models in the Rochester Epidemiology Project (REP) cohort.

REP (sex and SES^a^ group)	National-level ADI^b^, BER (95% CI)				State-level ADI, BER (95% CI)			
	RF^c^	LR^d^	SVM^e^	NB^f^	RF	LR	SVM	NB
Male								
High	0.2550^g^ (0.24-0.27)	0.2922^g^ (0.27-0.31)	0.3138^g^ (0.29-0.33)	0.3500^h^ (0.33-0.37)	0.2500^g^ (0.23-0.27)	0.2839^g^ (0.25-0.32)	0.3415^g^ (0.32-0.36)	0.3551^h^ (0.33-0.38)
Low	0.3001^h^ (0.21-0.39)	0.3113^h^ (0.23-0.40)	0.3378^h^ (0.25-0.44)	0.3165^g^ (0.24-0.40)	0.2756^h^ (0.24-0.32)	0.2943^h^ (0.27-0.33)	0.3458^h^ (0.31-0.38)	0.3302^g^ (0.29-0.36)
Female								
High	0.2606^g^ (0.24-0.28)	0.2988^g^ (0.28-0.32)	0.3266^g^ (0.31-0.35)	0.3585^g^ (0.34-0.37)	0.2335^g^ (0.21-0.26)	0.2815^g^ (0.26-0.30)	0.3341^g^ (0.32-0.35)	0.3682^h^ (0.35-0.39)
Low	0.2648^h^ (0.20-0.33)	0.3123^h^ (0.25-0.38)	0.3688^h^ (0.29-0.42)	0.4067^h^ (0.34-0.47)	0.2560^h^ (0.23-0.29)	0.2962^h^ (0.27-0.33)	0.3584^h^ (0.33-0.39)	0.3478^g^ (0.32-0.38)
Male and female								
High	0.2581^g^ (0.25-0.27)	0.2958^g^ (0.28-0.31)	0.3163^g^ (0.29-0.33)	0.3547^g^ (0.34-0.37)	0.2478^g^ (0.23-0.27)	0.2827^g^ (0.27-0.30)	0.3376^g^ (0.32-0.35)	0.3626^h^ (0.35-0.38)
Low	0.2772^h^ (0.23-0.33)	0.3124^h^ (0.27-0.36)	0.3437^h^ (0.29-0.39)	0.3752^h^ (0.33-0.42)	0.2637^h^ (0.24-0.29)	0.2955^h^ (0.28-0.32)	0.3547^h^ (0.33-0.38)	0.3411^g^ (0.32-0.36)

a SES: socioeconomic status.

b ADI: Area Deprivation Index.

c RF: random forest.

d LR: logistic regression.

e SVM: support vector machine.

f NB: Naïve Bayes.

g BER significantly lower.

h BER significantly higher.

Table 6 compares BERs within each AI-based dementia prediction model among SES-sex subgroups (using the same approach as Table 4) in the REP cohort; we selected “male in high SES” as the baseline category. From the table, most of both males and females with low SES exhibit higher BER compared to males with high SES. Furthermore, females with high SES, as indicated by the national-level ADI, exhibited higher BER compared to males with high SES. However, females with high SES, as indicated by the state-level ADI, exhibited the lowest BERs across almost all the evaluated models except for the NB model.

**Table 6.** Balanced error rate (BER) difference for male high socioeconomic status (SES) as a baseline for Table 5.

REP^a^ (sex and SES group)	National-level ADI^b^, BER				State-level ADI, BER			
	RF^c^	LR^d^	SVM^e^	NB^f^	RF	LR	SVM	NB
Male								
High	Base	Base	Base	Base	Base	Base	Base	Base
Low	Higher^g^	Higher^g^	Higher^g^	Lower^h^	Higher^g^	Higher^g^	Higher^g^	Lower^h^
Female								
High	Higher^g^	Higher^g^	Higher^g^	Higher^g^	Lower^h^	Lower^h^	Lower^h^	Higher^g^
Low	Higher^g^	Higher^g^	Higher^g^	Higher^g^	Higher^g^	Higher^g^	Higher^g^	Lower^h^

a REP: Rochester Epidemiology Project.

b ADI: Area Deprivation Index.

c RF: random forest.

d LR: logistic regression.

e SVM: support vector machine.

f NB: Naïve Bayes.

g Higher: BER significantly higher than baseline.

h Lower: BER significantly lower than baseline.

Table 7 presents a comparison of the relative differences in BERs between low and high SES groups, as indicated by both the ADIs and the HOUSES Index, across the 4 AI-based dementia prediction models in the MCSA cohort. The relative difference (value close to 0 is perfectly balanced between low and high SES groups), calculated as (low SES’ BER−high SES’ BER)/high SES’ BER, was first shown for the baseline case with no dataset balancing. Subsequently, the relative differences after balancing the training dataset (separately using national-level ADI, state-level ADI, and the HOUSES Index) are displayed. Within each model and SES measure, if the difference postbalancing was lower than the baseline, the corresponding cell is marked with “i”; otherwise, it is marked with “j”. As indicated by “h” in the table, balancing the training dataset using any one of the 3 SES measures was associated with narrower BER differences between high and low SES groups when stratified by that same SES measure. However, in several cases, most notably with the LR and RF models under national-level ADI balancing, the relative difference shifted from positive to negative. This shift indicates an inversion of the disparity, whereby the low SES group achieved a lower BER than the high SES group. Furthermore, when the training dataset was balanced using one SES measure but performance was stratified by another SES measure, BER differences were similarly reduced across nearly all cases. The exceptions occurred when SES was stratified by national-level ADI and the training dataset was balanced by state-level ADI or the HOUSES Index under the RF model or when the training dataset was balanced by national-level ADI but evaluated using the HOUSES Index with the SVM and NB models. For instance, balancing the training data on the HOUSES Index increased the BER disparity measured by the national-level ADI from 8.38% to 30.37% with the RF model. This pattern suggests that fairness interventions are not universally beneficial across different definitions of the protected attribute.

Table 8 presents the same comparison as in Table 7 but for the REP cohort. Similarly, as indicated by footnote “g” in the table, balancing the training dataset using any one of the 3 SES measures consistently reduced the BER differences between high and low SES groups when stratified by that same SES measure. A similar pattern of disparity inversion was observed, where balancing on the national-level ADI for the RF model and the state-level ADI for the SVM model also resulted in negative relative differences.

**Table 7.** Comparison of balanced error rate (BER) differences between low and high socioeconomic status (SES) groups under various data balancing strategies in artificial intelligence (AI)–based dementia prediction models (Mayo Clinic Study on Aging [MCSA] cohort).

MCSA (sex and balance on)	National-level ADI^a^, (low SES’ BER−high SES’ BER)/high SES’ BER (percentile)				State-level ADI, (low SES’ BER−high SES’ BER)/high SES’ BER (percentile)				HOUSES^b^ Index, (low SES’ BER−high SES’ BER)/high SES’ BER (percentile)			
	RF^c^	LR^d^	SVM^e^	NB^f^	RF	LR	SVM	NB	RF	LR	SVM	NB
Male and female												
N/A^g^	8.38%	33.63%	6.89%	34.71%	30.28%	15.57%	1.83%	6.98%	8.09%	10.95%	3.58%	17.63%
National-level ADI	−5.84%^hi^	−7.27%^hi^	3.49%^hi^	5.11%^hi^	2.53%^i^	2.14%^i^	1.13%^i^	2.94%^i^	4.27%^i^	9.79%^i^	5.90%^j^	18.13%^j^
State-level ADI	−30.37%^j^	−28.64%^i^	−5.31%^i^	−22.96%^i^	3.91%^hi^	1.11%^hi^	−0.27%^hi^	−1.12%^hi^	−1.95%^i^	−0.20%^i^	1.03%^i^	−9.75%^i^
HOUSES Index	25.13%^j^	−14.89%^i^	−2.33%^i^	1.15%^i^	15.89%^i^	2.45%^i^	1.52%^i^	−0.50%^i^	2.24%^hi^	3.43%^hi^	−0.86%^hi^	3.83%^hi^

a ADI: Area Deprivation Index.

b HOUSES: Housing-Based Socioeconomic Status.

c RF: random forest.

d LR: logistic regression.

e SVM: support vector machine.

f NB: Naïve Bayes.

g Not applicable.

h Results corresponding to models trained on datasets balanced using the same SES measure by which performance is stratified.

i Relative BER difference lower.

j Relative BER difference higher.

**Table 8.** Comparison of balanced error rate (BER) differences between low and high socioeconomic status (SES) groups under various data balancing strategies in artificial intelligence–based dementia prediction models (Rochester Epidemiology Project [REP] cohort).

REP (sex and balance on)	National-level ADI^a^,(low SES’ BER−high SES’ BER)/high SES’ BER [percentile])				State-level ADI, (low SES’ BER−high SES’ BER)/high SES’ BER [percentile])			
	RF^b^	LR^c^	SVM^d^	NB^e^	RF	LR	SVM	NB
Male and female								
N/A^f^	7.40%	5.61%	8.66%	5.78%	6.42%	4.53%	5.07%	−5.93%
National-level ADI	−1.80%^g^^h^	2.37%^g^^h^	3.28%^g^^h^	2.63%^g^^h^	−4.25%^h^	6.75%^i^	3.11%^h^	−2.70%^h^
State-level ADI	−4.14%^h^	9.26%^i^	−0.05%^h^	6.73%^i^	1.62%^g^^h^	0.16%^g^^h^	−0.52%^g^^h^	−3.28%^g^^h^

a ADI: Area Deprivation Index.

b RF: random forest.

c LR: logistic regression.

d SVM: support vector machine.

e NB: Naïve Bayes.

f Not applicable.

g Results corresponding to models trained on datasets balanced using the same socioeconomic status (SES) measure by which performance is stratified.

h Relative BER difference lower.

i Relative BER difference higher.

## Discussion

### Principal Findings

Our study demonstrates socioeconomic bias in AI-driven dementia prediction models. Across both the research cohort (MCSA) and the real-world cohort (REP), the combined male and female population with lower SES consistently exhibited higher BERs compared to their higher SES counterparts, except when using the NB model in the REP cohort. This discrepancy suggests that AI models, when trained on imbalanced SES datasets, may not generalize well across different socioeconomic groups, potentially reinforcing existing health care disparities. The findings highlight important differences in bias detection across SES measures. Both the ADI and the HOUSES Index were effective in identifying disparities, but they capture different aspects of socioeconomic disadvantage. The HOUSES Index, which is based on housing data, identified a more nuanced variation in model performance. In contrast, the ADI (both national-level and state-level rankings), which is derived from census block group–level data, showed broader disparities but may be less sensitive to individual economic conditions.

One important factor contributing to the observed differences in performance between high and low SES groups is the imbalance in their representation within both cohorts. Individuals from higher SES groups significantly outnumber those from lower SES groups. This skewed distribution can have a direct impact on model performance, as AI algorithms trained on predominantly high SES individuals may not adequately capture the health-related patterns of populations with low SES.

A key implication of this imbalance is that AI models become optimized for the majority (high SES) group, leading to lower accuracy and higher BERs for the understudied (low SES) group. Machine learning models inherently learn patterns based on the data they are exposed to, and if the majority of training samples come from high SES individuals, the model is likely to develop a bias toward their health characteristics and health care access patterns. This bias can manifest in the form of increased BER for participants with low SES. Furthermore, disparities in health care access and utilization between SES groups can compound these biases. Traditionally, it is argued that individuals from higher SES backgrounds have more comprehensive and consistent health records, whereas individuals with lower SES may experience gaps in care that lead to incomplete medical histories [23]. Our findings support this view: the low SES group exhibited a higher average number of years with missed visits compared to the high SES group, as reported in the Results section.

Another important observation is the interaction between SES and sex. In the MCSA cohort, high SES male participants generally exhibited the lowest BERs. In the REP cohort, however, the results depended on the specific SES measure used: high SES female individuals tended to have the lowest BERs when stratified by the state-level ADI, whereas this pattern was not observed with the national-level ADI. This discrepancy between cohorts may result from MCSA’s active recruitment and retention process, which likely selected for a subgroup of male participants with higher health literacy and more complete records than the general population—a selection bias less present in the passive data collection of the REP cohort. Furthermore, within the REP cohort, the fact that high SES female individuals did not exhibit this advantage under the national-level ADI highlights the differential validity of SES measures. Broader metrics such as the national-level ADI may fail to capture localized resource variations that drive health outcomes for female participants, nuances that are better reflected by more granular measures such as the state-level ADI.

To address these disparities, we applied the SMOTE-NC to balance the SES representation in the training data. This approach narrowed the observed SES-related disparities in BERs, demonstrating that balanced data distributions can improve AI model fairness. However, the effectiveness of this technique varied across SES measures—while balancing based on the HOUSES Index and state-level ADI consistently reduced BER disparities, national-level ADI balancing sometimes led to unintended increases in BER differences for certain models. This may indicate that the national-level ADI lacks the necessary resolution to distinguish SES levels within specific states effectively. Unlike the state-level ADI or the HOUSES Index, the national-level ADI may not adequately represent the local socioeconomic context required for effective data balancing. Furthermore, the limited concordance observed between the HOUSES Index and the ADI measures may explain why oversampling based on one measure resulted in disparities in the others.

While our balancing approach improved fairness in model performance for lower SES groups, it often came at the cost of a slight reduction in overall model performance. In some cases, this intervention led to an inversion of bias, resulting in the models performing better on the low SES group than the high SES group. The trade-off between fairness and predictive accuracy is a known challenge in AI-driven health care apps, and our results highlight this issue. Most balancing strategies led to improved performance for the low SES group while slightly decreasing overall predictive accuracy. However, an exception was observed in the MCSA cohort when balancing based on the HOUSES Index using LR, SVM, and NB, where both the high and low SES group performances improved. This suggests that housing-based SES metrics, such as the HOUSES Index, may be more effective for bias mitigation than area-level measures such as the ADI.

Beyond statistical significance, it is crucial to consider the clinical importance of these performance disparities. For instance, in the MCSA cohort, the NB model using the HOUSES Index yielded a BER of 39.3% for the high SES group compared to 46.2% for the low SES group. While this represents a modest absolute difference of 6.9 percentage points, its impact at a population level is substantial. In a hypothetical clinical screening scenario involving 1000 patients from each SES group, this disparity would translate to an additional 69 patients from low SES backgrounds being misclassified compared to their high SES counterparts. Such a systematic difference in accuracy, when deployed at scale, could further disadvantage already underserved populations in clinical care. Patients from socioeconomically disadvantaged backgrounds would be disproportionately subject to the consequences of misclassification, including delayed diagnosis and intervention in the case of false negatives, or unnecessary, costly, and anxiety-inducing follow-up procedures in the case of false positives. This quantification highlights how seemingly small statistical biases can manifest as meaningful clinical harms.

### Limitations

While this study provides valuable insights, it is important to recognize limitations. First, although our study cohorts (MCSA and REP) represent Midwestern populations with a dominant White demographic, they are not representative of the broader US population [39]. Therefore, the findings regarding SES- and sex-based bias warrant validation in more representative populations. Second, our AI models do not include certain important clinical factors, such as cognitive test scores, functional assessments, or advanced biomarkers, in dementia prediction. Instead, we focused on commonly available variables in real-world clinical settings because the primary goal of this study was to investigate AI bias rather than to maximize predictive accuracy. Third, relying on ICD codes to identify dementia cases in the REP cohort introduces potential ascertainment bias. In real-world settings, socioeconomic barriers to care may prevent low SES individuals from obtaining a formal diagnosis despite the presence of disease. Consequently, these undiagnosed individuals are liable to be misclassified as not having dementia. This specific form of label noise likely inflates the apparent error rates (specifically false negatives) for low SES groups independent of algorithmic performance. Fourth, the requirement for a 5-year observation window and subsequent follow-up may inadvertently exclude individuals with fragmented care. This selection factor suggests that the performance disparities reported here may underestimate the full extent of algorithmic bias. Additionally, while SMOTE-NC consistently reduced numerical BER disparities, the statistical significance of this reduction was not formally tested. These findings should be interpreted as descriptive evidence of fairness improvement. Finally, we did not consider temporal validation of the AI models over time, particularly in the REP cohort (real-world data). Health care systems are dynamic, and evolving diagnostic criteria for dementia and changes in ICD coding practices may lead to concept drift. Consequently, the models’ utility for predicting dementia risk in future patient populations requires further investigation. Nonetheless, our main objective was to assess the SES-driven AI model bias rather than to optimize prediction performance, and our findings successfully demonstrate the influence of SES on AI bias in dementia prediction.

### Future Work

In this study, BER was selected as the primary metric for evaluating model performance and fairness. This metric is particularly well suited for classification tasks with imbalanced classes, such as dementia prediction, because it gives equal importance to errors made on the understudied and majority classes, unlike standard accuracy. Our use of BER aligns with a fairness goal of achieving parity in overall error rates between demographic subgroups. However, it is important to acknowledge that “fairness” is a multifaceted concept, and no single metric can capture all its dimensions. Alternative fairness criteria could have been used, each with different implications. Future work should extend this analysis by evaluating these models against a broader suite of fairness metrics to provide a more holistic assessment of their performance trade-offs. We should also investigate whether differences in health care utilization patterns contribute to these variations, as female individuals may engage with health care services differently than male participants, potentially influencing AI model predictions.

Our further research should explore advanced fairness-aware algorithms that optimize both fairness and predictive accuracy, ensuring that model improvements do not disproportionately benefit or disadvantage any subgroup. Additionally, our future work should evaluate alternative debiasing strategies beyond oversampling, such as model recalibration and adversarial learning, to address structural biases inherent in the data.

### Conclusions

This study underscores the biases in AI models predicting dementia, with performance disparities observed across SES and sex groups. Individuals from lower SES backgrounds consistently experienced less accurate predictions, as reflected in higher BERs. These disparities suggest that AI models trained on real-world health care data may inadvertently reinforce patterns of underservice among certain populations, underscoring the need for fairness-aware modeling approaches.

Comparisons between SES measures revealed that the HOUSES Index and ADI capture different aspects of socioeconomic disadvantage, with the HOUSES Index potentially offering more granularity at the individual level. The observed sex-based disparities in model bias further emphasize the intersectionality of SDOH, highlighting the importance of multidimensional fairness evaluations in AI-driven health care applications.

Ultimately, this research reinforces the ethical imperative to integrate sociodemographic factors into AI model development and evaluation. Addressing these biases is essential for promoting fair AI-driven solutions in dementia risk prediction, where early and accurate diagnosis is critical for patient outcomes.

## Abbreviations

ADI: Area Deprivation Index
AI: artificial intelligence
BER: balanced error rate
DEM: dementia
HOUSES: Housing-Based Socioeconomic Status
LR: logistic regression
MCI: mild cognitive impairment
MCSA: Mayo Clinic Study on Aging
NB: Naïve Bayes
REP: Rochester Epidemiology Project
RF: Random Forest
SES: socioeconomic status
SMOTE-NC: Synthetic Minority Oversampling Technique for Nominal and Continuous features
SVM: support vector machine
